# Comparison of Endocrine Profile and *In Vitro* Fertilization Outcome in Patients with PCOS, Ovulatory PCO, or Normal Ovaries

**DOI:** 10.1155/2012/492803

**Published:** 2012-02-28

**Authors:** Yi-Ping Zhong, Ying Ying, Hai-Tao Wu, Can-Quan Zhou, Yan-Wen Xu, Qiong Wang, Jie Li, Xiao-Ting Shen, Jin Li

**Affiliations:** Reproductive Medicine Center, The First Affiliated Hospital, Sun Yat-sen University, Guangzhou 510080, China

## Abstract

*Aim*. To compare the basic endocrine profile and outcomes of *in vitro* fertilization (IVF) in women with polycystic ovary syndrome (PCOS), ovulatory polycystic ovaries (PCO), or normal ovaries (NO). *Methods*. The basic clinical features and *in vitro* fertilization and embryo transfer outcome in patients receiving IVF or intracytoplasmic sperm injection (ICSI) were retrospectively analyzed. *Results*. The body mass index, basal luteinizing hormone, and testosterone levels were significantly lower in patients with ovulatory PCO compared to those in patients with PCOS. The PCOS patients exhibited the shortest duration of ovarian stimulation and lowest dose of gonadotropin, followed by the ovulatory PCO and NO patients. The ovulatory PCO and PCOS patients showed similar levels of E2 on the human chorionic gonadotropin treatment day and numbers of oocytes, which were both significantly higher than those of the NO patients. The fertilization rate of the PCOS patients was significantly lower than the other two groups. Compared to NO patients, the cleavage rate was lower in both PCOS and ovulatory PCO patients, however, the number of available embryos was significantly more in these two groups. The incidence of the moderate to severe ovarian hyperstimulation syndrome (OHSS) was markedly higher in the PCOS and ovulatory PCO patients. *Conclusion*. Ovulatory PCO patients do not express similar endocrine abnormalities as PCOS patients. Although the fertilization rate and cleavage rate were relatively low in PCOS patients, ultimately, all the three groups showed similar transferred embryo numbers, clinical pregnancy rates, and implantation rates. Since the incidence of OHSS was much higher in the PCOS and ovulatory PCO patients, we should take more care of these patients and try to prevent severe OHSS.

## 1. Introduction

The latest advances in ultrasonic measurement techniques have helped indentify that 20~30% women have polycystic ovaries (PCO) [1]. The incidence of PCO is up to 34% for those women diagnosed by reproductive specialists [2]. A subpopulation of these PCO cases is ovulatory PCO [3], in which women do not display any typical symptom of the polycystic ovary syndrome (PCOS) although they do have PCO. Some ovulatory PCO patients reportedly show mild endocrine abnormalities including high levels of luteinizing hormone (LH) or androgen as well as insulin resistance, which are similar to those experienced by PCOS patients [4–6]. However, a recent study demonstrates that isolated PCO is an age-dependent, normal finding among ovulatory women that has no pathologic or clinical significance [7]. To date, numerous studies have extensively investigated the *in vitro* fertilization and embryo transfer (IVF-ET) in PCOS patients [8–13], whereas little information is available for IVF-ET in ovulatory PCO patients [14–17]. The aim of the present study was to directly address this deficiency in the literature and compare the ovulation and treatment of IVF-ET among patients suffering from PCOS, ovulatory PCO, or normal ovaries with other complications (NO).

## 2. Materials and Methods

### 2.1. Subjects

Patients receiving IVF or intracytoplasmic sperm injection (ICSI) treatment at the Reproductive Medicine Center of the First Affiliated Hospital, Sun Yat-sen University, between January and September 2010, were selected for analysis. Patient information was documented in details, including age, weight, menstruation history, pelvic ultrasound examination results, and basal hormone levels. Patients with endometriosis or any other endocrine complications such as thyroid dysfunction, hyperprolactinemia, cushing syndrome, or atypical congenital adrenal hyperplasia were excluded from the study. Based on the diagnosis, patients were divided into three groups: PCOS (122 patients, 122 cycles), ovulatory PCO (208 patients, 208 cycles), and normal ovaries (660 patients, 660 cycles).

There were 122 PCOS patients diagnosed according to the Rotterdam Standard who had a total of 122 IVF/ICSI cycles, Oligo-ovulation was defined as a menstrual cycle of longer than 35 days; hyperandrogenism was diagnosed with either clinical or biochemical profiles; PCO was defined as the presence of 12 or more follicles in each ovary measuring 2–9 mm in diameter, or increased ovarian volume larger than 10 mL. Moreover, 208 ovulatory PCO patients had 208 IVF/ICSI cycles, which were diagnosed with a regular menstrual cycle (21–35 days), no clinical or biochemical profiles of hyperandrogenism and the presence of 12 or more follicles with a diameter of 2–9 mm in either side of the ovary, and/or total ovary volume ≥10 mL. In addition, 660 patients with normal ovaries had a total of 660 IVF/ICSI cycles were recruited as controls, they all exhibited normal ovarian morphology and regular menstrual cycles. Causes of infertility for these patients include tubal blockage, pelvic adhesions, and/or male factor.

### 2.2. IVF-ET

Long-term pituitary downregulation was performed in all patients [18]. Briefly stated, long-acting gonadotropin-releasing hormone agonist 1.0 mg or 1.3 mg was intramuscularly administered in the midluteal phase (GnRH-a, Diphereline, France). Following pituitary downregulation, patients were administered gonadotropin (Gn, Gonal-F, Switzerland) from the 3th-5th day of the menstrual cycle. The initial Gn dose was determined by a variety of factors, including age, number of ovarian follicles, basal FSH levels, and history of ovarian response. During the ovarian stimulation, transvaginal ultrasound and serum sex hormone levels were monitored to evaluate the development of ovarian follicles, and the Gn dose was adjusted based on the intensity of ovarian response. When more than (and including) 2 ovarian follicles with the diameter ≥18 mm, or more than (and including) 3 ovarian follicles with the diameter ≥17 mm, were detected, patients were injected with 10,000 IU human chorionic gonadotropin (hCG, Switzerland) to trigger oocyte maturation. Oocytes were collected about 36 hours later. The fertilization protocol (regular IVF or ICSI) was determined by the condition of semen on the same day as the oocyte extraction. Embryo transplantation was determined by the number of oocytes, the estradiol (E2) level, and the patient conditions. No more than three embryos were implanted into the uterine cavity three days after the oocyte extraction. Patients started receiving hCG or progesterone treatment since the day of oocyte extraction. The urine and serum test of hCG was performed 14 days after embryo transfer. If the hCG test was positive, ultrasonic examination was conducted two weeks later to determine the clinical pregnancy.

### 2.3. Clinical Data Collection

A blood test was performed on the 2nd-5th day of the menstrual cycle before the treatment to determine the levels of FSH, LH, E2, and testosterone (T). The PCOS and ovulatory PCO patients received transvaginal ultrasound before the IVF treatment to examine the pelvic conditions. All ovulatory PCO patients and some of the PCOS patients exhibited the symptoms of polycystic ovary. During treatment, patient information was documented, including age, body mass index (BMI), duration of Gn administration, total Gn dose, E2 levels on the hCG day, endometrial thickness on the hCG day, numbers of collected oocytes, fertilization rates, oocyte cleavage rates, numbers of available embryos and transferred embryos, hCG positive rates, numbers of gestational sacs, incidence of spontaneous abortion, incidence of ovarian hyperstimulation syndrome (OHSS), and incidence of cycle cancellation. The OHSS classification was based on the “expert consensus on diagnosis and treatment of Polycystic Ovarian Syndrome” standard set by the Endocrinology Group of the Obstetrics and Gynecology Society, Chinese Medical Association (CMA).

### 2.4. Data Analysis

All data were represented in mean ± standard deviation (SD). The group differences were analyzed by analysis of variance (ANOVA) or rank test for quantitative data and Chi-square test for qualitative data. Statistical analyses were finished using SPSS 13.0 software. *P* < 0.05 was considered statistically significant with Bonferroni corrections.

## 3. Results

### 3.1. Basic Clinical Characteristics

Compared to the NO patients, patients with PCOS or ovulatory PCO were younger (*P* < 0.01). The PCOS patients exhibited the highest BMI as well as basal LH and T levels (*P* < 0.01), whereas the NO patients exhibited the highest basal FSH levels (*P* < 0.01) (Table 1).

### 3.2. Comparisons of Controlled Ovarian Hyperstimulation

Compared to the NO patients, patients with PCOS or ovulatory PCO exhibited a significantly shorter stimulation duration and lower Gn dose but showed much higher E2 levels on the hCG treatment day and more achieved oocytes (*P* < 0.01) (Table 2).

### 3.3. Comparisons of IVF-ET Outcome

The fertilization rate of the PCOS patients was significantly lower than the other two groups. The PCOS and ovulatory PCO patients exhibited similar lower cleavage rates, but more available embryos. We did not observe any significant differences in the numbers of transplanted embryos, pregnancy rates, clinical pregnancy rates, or implantation rates among the three groups. The PCOS patients exhibited a higher miscarriage rate (17.5%), although the difference was not significant. The incidence of moderate to severe OHSS was 15.8% and 11.1% for the PCOS and ovulatory PCO patients, respectively, which was significantly higher than that of the NO patients (5.4%, *P* < 0.01) (Table 3).

Besides moderate to severe OHSS, cycle cancellation was also a notable adverse outcome of the IVF treatment. Cycle cancellation could be classified into three types: (1) the cycle was canceled during the process of ovarian stimulation because of poor ovarian response; (2) the embryo transfer was canceled due to excessive numbers of oocyte achieved, high E2 levels on the hCG treatment day, and/or showing symptoms of overstimulation, including abdominal distension and apparent pelvic fluid; (3) other complications, including oocyte collection failure, abnormalities in the oocyte fertilization and/or cleavage, poor embryo quality, and/or endometrial factors. In the PCOS, ovulatory PCO, and NO patients, the rates of cycle cancellation due to poor ovarian response were 1.6% (2/122), 0% (0/208), and 2.7% (16/660), respectively; the rates of embryo transfer cancellation were 18.0% (22/122), 22.1% (46/208), and 4.9% (32/660), respectively; the rates of other causes were 1.6% (2/122), 1.0% (2/208), and 2.4% (16/660), respectively. Thus, the overall rates of cycle cancellation for these three groups were 21.2% (26/122), 23.1% (48/208), and 10.0% (64/660), respectively. Our results suggested that compared to the NO patients, the PCOS and ovulatory PCO patients exhibited significantly higher rates of embryo transfer cancellation and overall cycle cancellation (*P* < 0.05).

## 4. Discussion

Contrary to some reports [4–6], in the current study, we found that BMI and levels of LH and androgen were similar between the ovulatory PCO and NO patients, which were significantly higher than those in the PCOS patients. Insulin-sensitizing agent metformin which has been examined as a cotreatment during IVF in women with PCOS, brought an increase in pregnancy rate [19] and a significant reduction in rates of OHSS [20], however, for women with PCO but no other manifestations of PCOS, metformin cotreatment before and during IVF did not bring any positive effect in clinical pregnancy, live birth, or severe OHSS [21]. The most likely explanation is that women with PCO may be less insulin resistance compared with women with PCOS. While some women with PCO to share some endocrinological abnormalities with those with PCOS, the difference between these studies may be attributed to variations in categorizing patients. According to the widely used Rotterdam diagnostic criteria for PCOS, ultrasonic diagnosis of PCO is an important, but not essential characteristic of PCOS. Thus, PCO and PCOS are not necessarily related to each other. In this study, the ovulatory PCO patients did not have the endocrine and metabolic abnormalities manifested by the PCOS patients, indicating that polycystic ovary might only represent some normal variation in the ovarian morphology.

All patients in the present study were treated with standard protocol of long-term GnRH-a administration in midluteal phase, which was commonly used for infertility women including PCOS patients. In addition to its well-known advantages such as easy to operate and satisfactory pregnancy rates, for PCOS patients, GnRH-a can effectively reduce the LH/androgen level and inhibit the inflammatory factors, thus may improve the quality of the oocytes and embryos as well as endometrial receptivity. Compared with the PCOS patients treated with oral contraceptive pill [OCP] alone, patients treated with OCP plus long-term GnRH agonist exhibit lower hormone levels and better amelioration of clinical symptoms [8–10]. In addition, the fertilization rates and pregnancy rates of GnRH-a-treated PCO patients were similar to those of the non-PCO patients [14]. Therefore, based on the current findings, clinical experience, as well as our findings in the present study, we propose that GnRH-a long-term protocol is suitable strategy for the PCOS patients treated with IVF.

Our study showed that both the ovulatory PCO and PCOS patients displayed high-ovarian responsiveness, which was consistent with previous research [15]. Compared to the NO patients, the ovulatory PCO and PCOS patients exhibited reduced duration of ovarian stimulation and total Gn dose, as well as increased E2 levels and number of collected oocytes. Previous studies have suggested that compared to other infertility patients, PCOS patients exhibited a higher degree of ovary vascularization during the process of ovarian stimulation, which paralleled with vascular endothelial growth factor [VEGF] levels in the serum and follicular fluid. The ovary vascularization and VEGF levels were positively correlated with the E2 levels and numbers of oocytes achieved [11, 22]. In addition, VEGF can enhance the proliferation and function of granulosa cells [23]. Coffler et al. [12] suggest that in PCOS patients, follicular granulosa cells can show two types of response to FSH: (1) PCOS patients show remarkably higher E2 levels in response to FSH above the threshold dose; (2) PCOS patients treated with a single FSH stimulation exhibit much faster rise and decay of the E2 levels, which were much different from the response of the control patients. However, serum FSH levels of both groups were similar, a phenomenon which is probably explained by the fact that most follicular granulosa cells have either apoptosed or cannot effectively respond to FSH.

Based on previous and current findings, we conclude that both PCOS and ovulatory PCO patients are sensitive to Gn stimulation, thus it is difficult to determine an appropriate Gn dose for each individual patient. In our study, although we gradually reduced the Gn dose to avoid such overstimulation, the follicle degeneration and atresia were still observed in some patients, which were also reported by Coffler et al. [12]. Further work is needed to address this issue.

Compared to the NO patients, the PCOS but not the ovulatory PCO patients showed lower fertilization rates, whereas both groups exhibited a significantly lower oocyte cleavage rates and more available embryos. Thus, we further investigated whether the lower fertilization rates of the PCOS patients were due to abnormal oocyte morphology or changes in cytosolic factors which could affect the quality of the oocytes. Previous studies report that the morphology of the cytoplasm and extra cellular matrix of the oocytes and embryos were similar among the PCOS, PCO, and NO patients, indicating that the affected fertilization and early embryonic development of the PCOS and/or PCO patients are not because of intrinsic abnormalities of the oocytes [24].

Endocrine disorders and internal oocyte abnormalities can also result in low rates of oocyte fertilization and cleavage. For instance, high LH levels and insulin resistance can cause the malfunction of follicular granulosa cells and the abnormal expression of GDF-9, both of which markedly reduce the quality of the embryos [3, 14, 25].

Our results also demonstrated that the ovulatory PCO and PCOS patients exhibited similar rates of pregnancy, clinical pregnancy, and implantation. Although the higher average age of the NO patients might be a confounding factor for the analysis, we still considered that the pregnancy rates of the ovulatory PCO and PCOS patients were satisfactory. Our observations were consistent with previous studies by Esmailzadeh et al. [14], Kim et al. [15], Swanton et al. [16], and Esinler et al. [17]. In Esmailzadeh et al., the fertilization and pregnancy rates of the ovulatory PCO patients treated with GnRH-a long-term protocol were similar to those of the non-PCO patients [14]. Similar outcomes (duration of stimulation, dose of Gn used, E2 level on hCG day, number of retrieved oocytes, rates of implantation, clinical pregnancy and miscarriage, any incidence of severe OHSS) were observed in patients with PCOS and sonographic PCO-only for IVF-ET treatment [15]. Live birth rates are similar among women with PCO (38%), PCOS (37%), and normal ovaries (40%). Severe OHSS rates were significantly higher in women with PCO (12.6%) and PCOS (15.4%) compared to those with normal ovaries (2.7%) [16]. In the study of Esinler et al., the satisfactory pregnancy rates of the ovulatory PCO and PCOS patients were due to sufficient numbers of collected oocytes, fertilized oocytes, and transferred high-quality embryos [17].

PCOS patients exhibited the highest abortion rate, although no significant difference was detected, which could be explained by small sample size and age variation. Whether this high risk of abortion is related to the quality of the oocytes remains unclear. The risk may as well be caused by other factors than the oocytes. For instance, central obesity patients exhibit high LH levels and insulin resistance which can cause abortion, whereas administration of GnRH-a to these patients can effectively reduce the risk of abortion [24]. Wang et al. [13] postulated that the relatively high abortion rates in the PCOS patients were due to combined effects of obesity, treatment for infertility, and causes of infertility. After correction for these factors, the abortion rates in the PCOS patients were comparable to those in the controls, indicating that the intrinsic factors of PCOS contributed little to the abortion issue. In addition, the high E2 levels induced by ovary stimulation might increase the risk for abortion by lowering the endometrial capacity. However, both the viewpoints need further research for verification.

In summary, a subpopulation of polycystic ovaries cases are called ovulatory PCO patients, who do not display high levels of androgen and have regular menstrual cycles although they do have PCO. Unlike the PCOS patients, they have almost normal endocrine and metabolic characteristics and their PCO may be a normal variation of ovary morphology. When treated with IVF-ET, ovulatory PCO and PCOS patients similarly manifest high ovarian responsiveness, relatively good IVF pregnancy rates, and incidence of adverse outcomes, including high OHSS risk, cycle cancellation rates and abortion rates (although the differences were not statistically significant).

## Figures and Tables

**Table 1:** Comparisons of basic clinical features (mean ± SD).

Parameter	PCOS	Ovulatory PCO	NO	*P*-value
Age (year)	30.8 ± 3.8 (21 − 42)	30.6 ± 3.9 (22 − 41)^c^	32.4 ± 5.7 (21 − 45)^b^	0.000
BMI	22.6 ± 3.4^a^	20.7 ± 2.4	20.8 ± 2.7^b^	0.000
Basal FSH (IU/L)	5.1 ± 1.6	4.9 ± 1.4^c^	6.4 ± 1.7^b^	0.000
Basal LH (IU/L)	7.3 ± 3.7^a^	5.6 ± 2.1	5.4 ± 1.9^b^	0.000
Basal E2 (pg/mL)	39.9 ± 22.1	36.8 ± 16.8	38.3 ± 21.4	0.772
Testosterone (ng/mL)	1.16 ± 0.57^a^	0.61 ± 0.43	0.64 ± 0.33^b^	0.000

Note: a: PCOS versus PCO; *P* < 0.01; b: PCOS versus NO; *P* < 0.01; c: PCO versus NO; *P* < 0.01.

**Table 2:** Comparisons of controlled ovarian hyperstimulation (mean ± SD).

Parameter	PCOS	Ovulatory PCO	NO	*P*-value
Gn duration (day)	10.2 ± 1.3^a^	10.5 ± 1.8^c^	11.3 ± 2.6^b^	0.000
Total Gn dose (IU)	1843 ± 560^a^	1975 ± 621^c^	2227 ± 765^b^	0.000
E2 (hCG day) (pg/mL)	3071 ± 2637	3162 ± 2716^c^	2465 ± 1879^b^	0.000
Endometrial thickness (HCG day) (mm)	11.3 ± 3.5	10.8 ± 2.3	10.9 ± 2.6	0.262
Number of oocytes	16.5 ± 8.6	17.1 ± 7.4^b^	11.7 ± 6.9^c^	0.000

Note: a: PCOS versus PCO; *P* < 0.01; b: PCOS versus NO; *P* < 0.01; c: PCO versus NO; *P* < 0.01.

**Table 3:** Comparisons of IVF-ET outcome.

Parameter	PCOS	Ovulatory PCO	NO	*P*-value
% of fertilization (IVF cycle)	69.2 (950/1373)^a^	76.9 (1797/2338)	78.6 (6223/7922)^b^	0.000
% of oocyte cleavage	87.4 (830/950)	86.1 (1547/1797)^c^	91.8 (5713/6223)^b^	0.000
Available embryos	9.3 ± 5.9	10.6 ± 5.7^c^	6.3 ± 4.2^b^	0.000
Transferred embryos	2.2 ± 0.5	2.3 ± 0.5	2.3 ± 0.6	0.723
% of pregnancy	51.0 (50/98)	53.2 (67/126)	47.2 (274/580)	0.423
% of clinical pregnancy	40.8 (40/98)	43.7 (55/126)	39.3 (228/580)	0.667
% of implantation	25.5 (56/220)	26.0 (75/288)	24.7 (328/1328)	0.878
% of miscarriage	17.5 (7/40)	10.9 (6/55)	8.8 (20/228)	0.244
% of OHSS	15.8 (19/120)	11.1 (23/208)^c^	5.4 (34/626)^b^	0.000

Note: a: PCOS versus PCO; *P* < 0.01; b: PCOS versus NO; *P* < 0.01; c: PCO versus NO; *P* < 0.01.
